# Rare Presentation of Pediatric Nervous System Lyme Disease: A Case Report

**DOI:** 10.7759/cureus.60535

**Published:** 2024-05-18

**Authors:** Ia Khurtsilava, Darejan kanjaradze, Natia Tsirdava, Tsitsino Parulava, Irakli Darsania

**Affiliations:** 1 Pediatrics, Tbilisi Pediatric Private Clinic, Tbilisi, GEO; 2 Pediatrics, Petre Shotadze Tbilisi Medical Academy, Tbilisi, GEO; 3 Pediatric Intensive Care Unit, Tbilisi Pediatric Private Clinic, Tbilisi, GEO; 4 Pediatric Surgery, Tbilisi Pediatric Private Clinic, Tbilisi, GEO

**Keywords:** pediatric infectious disease, lyme neuroborreliosis, lyme borreliosis, nervous system lyme disease, lyme's disease

## Abstract

Lyme disease is a multisystem infectious disease. It is caused by the dissemination of *Borrelia spirochetes* after a tick bite. It has various manifestations across different age groups. Commonly involved organs are the skin, joints, and nervous system. Nervous system Lyme disease has a wide spectrum of manifestations. While facial nerve palsy and subacute meningitis are commonly observed in the pediatric population, our case report reveals an uncommon manifestation of the nervous system Lyme disease. A four-year-old patient exhibited mood changes, behavioral issues, and generalized tonic-clonic seizures. Extensive diagnostic workup initially yielded no clear cause until positive IgM and IgG serology for *Borrelia* suggested Lyme neuroborreliosis. This differs from the usual symptoms seen in pediatric cases. The patient responded positively to antibiotic treatment, but persistent post-treatment behavioral issues raised questions about potential long-term effects. This case underscores the importance of considering Lyme disease in atypical presentations, even in non-endemic areas, necessitating an adaptable diagnostic approach for improved outcomes, especially in pediatric patients. Continued research into the comprehensive understanding of Lyme disease in pediatric patients is crucial.

## Introduction

Lyme borreliosis is a multi-organ infectious disease initiated by the bite of infected ticks. Predominantly recognized as the most common tick-borne infection in Europe, the United States, and Canada, Lyme disease is caused by various genospecies. Three species are most prevalent in the world: *Borrelia burgdorferi* is the most common cause in North America while *B. afzelli* and *B. garinii *are the predominant species in Asia and Europe [1]. The incidence of Lyme disease In Europe is notably high in endemic areas, particularly in Austria, Sweden, Switzerland, Norway, Slovenia, and the Netherlands, where it exceeds 100 cases per 100,000 individuals [2,3]. Conversely, Georgia is not traditionally classified as an endemic area for Lyme disease, and data on its prevalence, particularly in pediatric populations, is deficient.

Lyme disease manifests in three stages: early localized, early disseminated, and late persistent, each affecting different organs of the body, including the skin, joints, heart, and nervous system [3,4]. The early localized form often presents with erythema migrans, a characteristic red macula, that evolves into a ring-shaped patch with marked linings following a tick bite [2-4].

While migratory and recurrent arthralgias may emerge as early manifestations, Lyme arthritis can develop months later. The neurotropic nature of *Borrelia* spirochetes can lead to the dissemination of Lyme borreliosis into the nervous system, resulting in a condition known as the nervous system Lyme disease. In children, the most common manifestations of neuroborreliosis include facial nerve palsy and aseptic meningitis. The variations in symptoms observed in different geographical regions and age groups are likely due to differences in the types of *Borrelia* species [3-9].

This case of Lyme neuroborreliosis in a non-endemic area emphasizes the need to stay alert and consider Lyme disease in regions not typically linked with the disease. It also highlights the complex clinical symptoms that can occur in various stages and affect different organ systems.

## Case presentation

A four-year-old boy was brought to Tbilisi Pediatric Private Clinic by ambulance after experiencing generalized tonic-clonic seizures that did not improve with one dose of diazepam therapy given by the ambulance. Over the last two days, the parents observed an increase in the boy's fatigue, a refusal to walk, and two instances of falling. The mother reported that the child had briefly lost consciousness three times and he couldn't recall the episode. The morning of the presentation, the child woke up feeling lethargic, followed by tonic-clonic seizures, and loss of awareness. The child was previously healthy. There was no recent traveling and no sick contacts. Immunizations were up-to-date. 

Upon arrival, vital signs were recorded as follows: temperature at 36.8°C, pulse at 140 beats per minute, respiratory rate (RR) at 10 breaths per minute, and oxygen saturation (SpO2) at 63% room air. The skin was cyanotic, extremities felt cool, capillary refill time was >3 seconds, breathing was irregular, pulses were weak, and pupils were mildly mydriatic. Abdominal examination showed no distension or hepatosplenomegaly. Despite the administration of rectal diazepam twice for ongoing seizures, there was no improvement. The condition was identified as status epilepticus. The patient was intubated and intravenous midazolam infusion was initiated. Intravenous normal saline solutions were administered as a bolus. Initial laboratory studies were conducted (Table 1). Blood gas analysis showed pH 6.9, base excess 13.3 mmol/L, partial pressure of carbon dioxide (PCO2) 102.4 mmHg, bicarbonate (HCO3) 14.6, and anion gap 17 mmol/L. Electrolytes were sodium (Na) 141 mmol/L, potassium (K) 3.3 mmol/L mEq/L, and chloride (Cl) 106 mmol/L.

**Table 1:** Complete blood count and C-reactive protein

Test name	Initial	One week after treatment	Unit	Reference range
Hemoglobin (HB)	12.7	11.9	g/dL	10.6-13.2
Red Blood Cells (RBC)	4.6	4.3	x 10^12^/L	3.9-4.96
White Blood Cells (WBC)	13.6		x 10^9^/L	4.27-11.40
Platelets	469	324	x 10^9^/L	199-400
Granulocytes	33.9%		-	
Agranulocytes	61.1%.		-	
C -reactive protein	19.2	0.97	mg/dl	<6

**Table 2:** CSF findings HSV: herpes simplex virus; PCR: polymerase chain reaction

	Patient values		Reference values
Cells, mm^3^	2		< 3/mm^3^
Proteins, mg/dL	0.256		0.22-0.33 g/L
Glucose, mg/dL	4.82		2.8–3.9 mmol/l
HSV-1 and HSV-2 DNA detection (by PCR)	Negative		–

Additional tests included a chest X-ray, which showed no abnormalities, and a CT scan of the head, revealing no changes. PCR tests for cytomegalovirus (CMV), herpes simplex virus (HSV) I, and HSV II in CSF were negative (Figure 2). Urine analysis showed light yellow, gravity 1015, pH 7, ketones +, WBC 4-5, nitrites negative. Total protein was 61.4 g/L, urea was 1.8 mmol/L, and liver function tests showed aspartate aminotransferase (AST) 27 U/L, alanine transaminase (ALT) 8.6 U/L, and albumin 36.5 g/L. Coagulation factors were normal. Enzyme-linked immunosorbent assay (ELISA) for *Morbillivirus* IgM (measles) was 0.079 (second sample also negative). No epileptic patterns were identified in EEG. A prolonged EEG, incorporating sleep deprivation and hyperventilation, was suggested for further evaluation.

Ceftriaxone and acyclovir were commenced as part of the treatment plan. Acyclovir was discontinued following the second negative result for the HSV.

Following the initial assessment, no apparent cause was identified. The patient exhibited slight clinical improvement. Despite non-classical manifestations, Lyme disease was considered and serum IgM and IgG serology (ELISA) was conducted (Table 3). This was followed by positive Western blot assay and quantification of virus-specific antibodies in CSF and diagnosis of Lyme disease was confirmed. Ceftriaxone therapy was changed to doxycycline. 

**Table 3:** Serology for Borrelia (ELISA)* *Euroimmun, Lübeck, Germany

Serum	Patient values	Reference
IgG	0.3	0.8-1.1
IgM	2.7	0.8-1.1
Western Blot *Borrelia burgdorferi; *Western Blot – Ig G Negative, Western Blot – Ig M Positive		

Upon discussing the results with the parents, they recalled noticing a rash four months ago, which resolved on its own without medical intervention, but the mother couldn't describe the rash clearly. 

After one week of treatment with antibiotics, the follow-up CBC tests showed no abnormalities. Other tests, including blood gases, electrolytes, urine tests, and liver enzymes, also remained within normal ranges. The patient was discharged and continuously monitored. Doxycycline was recommended for an additional two weeks, and the patient returned for a follow-up visit. While there was improvement in the clinical condition, the child exhibited mood changes and sleep disorders with night terrors. The mother reported the presence of imaginary friends and anxiety.

Four months after the initial diagnosis, severe behavioral problems persisted, including frequent tantrums, screaming, and hallucinations. The patient received psychiatric and psychological support throughout this period. After six months, the patient underwent reevaluation, revealing significant improvement. The boy exhibited mild mood swings.

## Discussion

Lyme borreliosis is caused by spirochetes of the *B. burgdorferi *sensu lato complex, primarily *B. burgdorferi* sensu stricto, *B. afzelii*, and *B. garinii *[1-3]. Neuroborreliosis presents with symptoms such as facial nerve palsy, meningitis, and radiculopathy, with variations between European and American populations due to different *Borrelia* species. Symptoms may manifest weeks to months after exposure, with early Lyme neuroborreliosis typically having a short duration in children [3,5,7]. However, diagnosing Lyme neuroborreliosis in children, particularly in non-endemic areas, is challenging due to its rarity and nonspecific manifestations. The limited availability of specific Lyme disease tests further complicates diagnosis in non-endemic areas.

In endemic regions, Lyme disease is often diagnosed clinically based on the patient's history and the development of symptoms. However, if there is a suspicion of neuroborreliosis, the American Academy of Neurology recommends evaluating for *Borrelia*-specific antibodies, and histopathologic and microbiologic evidence of *B. burgdorferi* [5,8]. This typically involves detecting *Borrelia*-specific antibodies in CSF, which is crucial for confirming the diagnosis of neuroborreliosis. Direct detection of *B. burgdorferi* can also be performed, but it is rarely utilized due to its low sensitivity, long incubation period, and the requirement of special culture media. Additional CSF findings may include a lymphocytic pleocytosis, resembling that of aseptic meningitis [5].

While various recommendations exist for treating neuroborreliosis in children, the key is to approach each patient’s case individually based on clinical evidence. Clinicians must consider factors such as age, allergies, and underlying conditions to determine the most suitable treatment option. Current guidelines suggest several medications for managing both early and late Lyme neuroborreliosis, including penicillin G, ceftriaxone, cefotaxime, or doxycycline [10]. However, the specific dosage, frequency, route of administration, and duration of treatment remain topics of debate [10,11].

Treatment options for pediatric Lyme disease vary based on the severity of the infection and the patient's age [10,11]. For early uncomplicated Lyme disease, outpatient oral therapy with doxycycline is recommended [10]. In children under eight years of age, amoxicillin is the preferred choice. In cases of more severe infection, such as neuroborreliosis, intravenous (IV) ceftriaxone is typically preferred due to its favorable dosing schedule [11]. However, IV cefotaxime and IV penicillin G have also been used successfully [10,11]. Doxycycline is not recommended in children under eight years of age due to the risk of dental staining, although some evidence suggests benefits may outweigh risks, as seen in our case where doxycycline led to significant clinical improvement [5,8].

Monitoring the clinical manifestations is crucial for assessing the effectiveness of treatment. In the pediatric population, the neuropsychological prognosis is generally positive, with long-term neuropsychologic disorders being uncommon compared to adults who may experience cognitive difficulties and persistent neurological symptoms. However, approximately one-fifth of children with Lyme neuroborreliosis may develop sensory or motor sequelae [5,8,10].

In our case, the patient experienced prolonged and severe behavioral problems, including frequent tantrums, screaming, and hallucinations. The patient received psychiatric and psychological support during this period, and these symptoms gradually resolved after six months. We were unable to find a case report with similar manifestations to our patient's. However, data presented at a Riga Stradiņš University (Riga, Latvia) conference demonstrated an atypical manifestation of Lyme disease in a five-year-old boy with afebrile seizures [12]. The authors state that the awareness of uncommon clinical manifestations in pediatric patients with Lyme neuroborreliosis is crucial for clinicians to successfully diagnose and treat the disease.

Although neuroborreliosis is rare in the pediatric population, Georgia is not considered an endemic area for Lyme disease, and our patient's manifestations do not align with typical presentations of neuroborreliosis, we believe that this case underscores the importance of considering Lyme borreliosis in the differential diagnosis when complex neurological symptoms arise in pediatric patients.

## Conclusions

This case underscores the diagnostic challenges associated with pediatric neurological symptoms and emphasizes the need for a thorough and flexible diagnostic approach. Considering Lyme disease as a potential cause of pediatric neurological problems despite the absence of typical manifestations is particularly challenging in non-endemic areas. we believe that this case might provide a basis for the implementation of new guidelines, including differential diagnosis for neurological symptoms in non-endemic areas. The prolonged post-treatment behavioral problems raise questions about the potential long-term effects of neuroborreliosis and show the need for continued research into a comprehensive understanding of Lyme disease in pediatric patients, especially in non-endemic areas where late diagnosis can be a major issue.

## References

[1] Burn L, Vyse A, Pilz A (2023). Incidence of Lyme borreliosis in Europe: a systematic review (2005-2020). Vector Borne Zoonotic Dis.

[2] Ogrinc K, Lotrič-Furlan S, Maraspin V, Lusa L, Cerar T, Ružič-Sabljič E, Strle F (2013). Suspected early Lyme neuroborreliosis in patients with erythema migrans. Clin Infect Dis.

[3] Lantos PM, Rumbaugh J, Bockenstedt LK (2021). Clinical practice guidelines by the Infectious Diseases Society of America (IDSA), American Academy of Neurology (AAN), and American College of Rheumatology (ACR): 2020 guidelines for the prevention, diagnosis and treatment of Lyme disease. Clin Infect Dis.

[4] Guet-Revillet H, Levy C, Vallet C (2019). Lyme neuroborreliosis in children: report of nine cases and a review of the literature. Arch Pediatr.

[5] Luft BJ, Steinman CR, Neimark HC (1992). Invasion of the central nervous system by Borrelia burgdorferi in acute disseminated infection. JAMA.

[6] Bruinsma RA, Zomer TP, Skogman BH, van Hensbroek MB, Hovius JW (2023). Clinical manifestations of Lyme neuroborreliosis in children: a review. Eur J Pediatr.

[7] Gern L, Pierre-Francois H (2002). Ecology of Borrelia burgdorferi sensu lato in Europe. Lyme Borreliosis: Biology, Epidemiology and Control.

[8] Kozak S, Kaminiów K, Kozak K, Paprocka J (2021). Lyme neuroborreliosis in children. Brain Sci.

[9] Estrada-Peña A, Cutler S, Potkonjak A (2018). An updated meta-analysis of the distribution and prevalence of Borrelia burgdorferi s.l. in ticks in Europe. Int J Health Geogr.

[10] Meissner HC, Steere AC (2022). Management of pediatric Lyme disease: updates from 2020 Lyme guidelines. Pediatrics.

[11] Kaciński M, Zajac A, Skowronek-Bała B, Kroczka S, Gergont A, Kubik A (2007). CNS Lyme disease manifestation in children. Przegld Lekarski.

[12] Senica SO, Šantová A, Gazali V, Zobel J, Gallistl S (2022). Atypical Presentation of Pediatric Lyme Neuroborreliosis: A Case Report [Conference Paper]. https://www.researchgate.net/publication/360612489_Atypical_presentation_of_pediatric_Lyme_neuroborreliosis_a_case_report.

